# 5-FU targets rpL3 to induce mitochondrial apoptosis via cystathionine-β-synthase in colon cancer cells lacking p53

**DOI:** 10.18632/oncotarget.10385

**Published:** 2016-07-02

**Authors:** Valentina Pagliara, Assunta Saide, Emma Mitidieri, Bianca Roberta d'Emmanuele di Villa, Raffaella Sorrentino, Giulia Russo, Annapina Russo

**Affiliations:** ^1^ Department of Pharmacy, University of Naples “Federico II”, 80131 Naples, Italy

**Keywords:** nucleolar stress, p53, CBS, colon cancer, apoptosis

## Abstract

Recent findings revealed in cancer cells novel stress response pathways, which in response to many chemotherapeutic drugs causing nucleolar stress, will function independently from tumor protein p53 (p53) and still lead to cell cycle arrest and/or apoptosis. Since it is known that most cancers lack functional p53, it is of great interest to explore these emerging molecular mechanisms. Here, we demonstrate that nucleolar stress induced by 5-fluorouracil (5-FU) in colon cancer cells devoid of p53 leads to the activation of ribosomal protein L3 (rpL3) as proapoptotic factor. rpL3, as ribosome-free form, is a negative regulator of cystathionine-β-synthase (CBS) expression at transcriptional level through a molecular mechanism involving Sp1. The rpL3-CBS association affects CBS stability and, in addition, can trigger CBS translocation into mitochondria. Consequently apoptosis will be induced through the mitochondrial apoptotic cell death pathway characterized by an increased ratio of Bax to Bcl-2, cytochrome c release and subsequent caspase activation. It is noteworthy that silencing of CBS is associated to a strong increase of 5-FU-mediated inhibition of cell migration and proliferation. These data reveal a novel mechanism to accomplish p53-independent apoptosis and suggest a potential therapeutic approach aimed at upregulating rpL3 for treating cancers lacking p53.

## INTRODUCTION

Colorectal cancer represents the third most frequently diagnosed malignancy in the world [1]. Despite recent advances in chemotherapy, currently used anticancer molecules are unable to improve the prognosis of advanced or recurrent colon cancer, which remains incurable. The anti-metabolite agent 5-FU is a widely used chemotherapeutic drug for the treatment of a variety of solid tumors [2] and it remains the standard first-line drug for the treatment of colon cancer [3]. Recently, some evidences indicate that 5-FU is able to induce ribosomal stress and disruption of the nucleolus with the consequent release of some ribosomal proteins that, exerting extra-ribosomal functions [4], in turn activate p53 and its target p21. It is known that most cancer cells contain mutant p53 or null p53 [5]. p53 mutations have been described in 70% of colon cancer [6]. Several studies have demonstrated that the loss of p53 function reduced cellular sensitivity to 5-FU [2]; however, the molecular mechanism by which this occurs is still a matter of debate [7]. Therefore, a better understanding of the p53 independent molecular mechanisms of 5-FU effects on cancer cells could contribute to improve the therapy of colon cancer. Recently, we have demonstrated that human rpL3 is able to activate the transcription of p21 in a p53-independet manner [8]. In addition, we have demonstrated that rpL3 acts as a stress sensing molecule essential for cell response to 5-FU treatment in colon cancer cells lacking active p53. In particular, our results indicated that rpL3 overexpression is able to improve the cytotoxic effects of 5-FU, which acts as DNA damage agent inducing apoptosis. Conversely, the loss of rpL3 makes chemotherapeutic effects of this drug ineffective [9, 10].

Clinical and epidemiological studies have evaluated the relationship between hydrogen sulfide (H_2_S) and colon cancer demonstrating that, in cancer cells a molecular switch comprised of H_2_S and Nicotinamide phosphoribosyltransferase (Nampt) is responsible for modifications of phenotype, of gene expression pattern and dedifferentiation [11, 12].

H_2_S intracellular biosynthesis is enzymatically regulated. It is generated from L-cysteine by two pyridoxal-5′-phospate–dependent (PLP) enzymes, cystathionine-β-synthase (CBS) and cystathionine-γ-lyase (CSE), and in PLP independent manner by the combined action of cysteine aminotransferase and 3-mercaptopyruvate sulfurtransferase (3-MST) [13].

It has been recently demonstrated that CBS is abundantly expressed in human colon cancer cell lines and in human colon cancer tissue specimens, resulting in increased H_2_S production [14]. CBS activity is tightly regulated at the transcriptional level [15]. Among the transcription factors, Sp1 represents a key regulator involved in the control of CBS transcription [16]. We have previously demonstrated that free rpL3 associates to Sp1 modulating the transcriptional factor activity [8].

Given these observations, in the attempt to characterize the molecular mechanism underlying rpL3-mediated cell response to 5-FU chemotherapy in colon cancer, we have investigated the existence of a regulatory mechanism of CBS expression mediated by rpL3. Here we provide for the first time evidences supporting the existence of a new extraribosomal function of rpL3, that is its regulatory role on the expression of the cytosolic enzyme CBS. In particular we demonstrate that in presence of 5-FU induced nucleolar stress, ribosome free rpL3 is able to: i) downregulate Sp1-mediated CBS transcription; ii) trigger mitochondrial translocation of CBS; iii) modify CBS protein stability; iv) induce mitochondrial apoptosis mediating the increase of Bax/Bcl-2 ratio, cytochrome c release and caspase activation.

## RESULTS

### Role of rpL3 in the CBS/H_2_S axis in colon cancer cells

In order to identify the rpL3 molecular targets that mediate its pro-apoptotic function upon drug-induced nucleolar stress [8–10], we became interested to assess the intracellular levels of CBS after 5-FU treatment in human colon cancer cells devoid of p53. To this aim, HCT 116^p53−/−^ cells and rpL3ΔHCT 116^p53−/−^ cells, a cell line stably depleted of rpL3, were treated with 100 μM 5-FU for 24 h. After the treatment, the cells were lysated and protein extracts were analyzed by western blotting.

**Figure 1:**
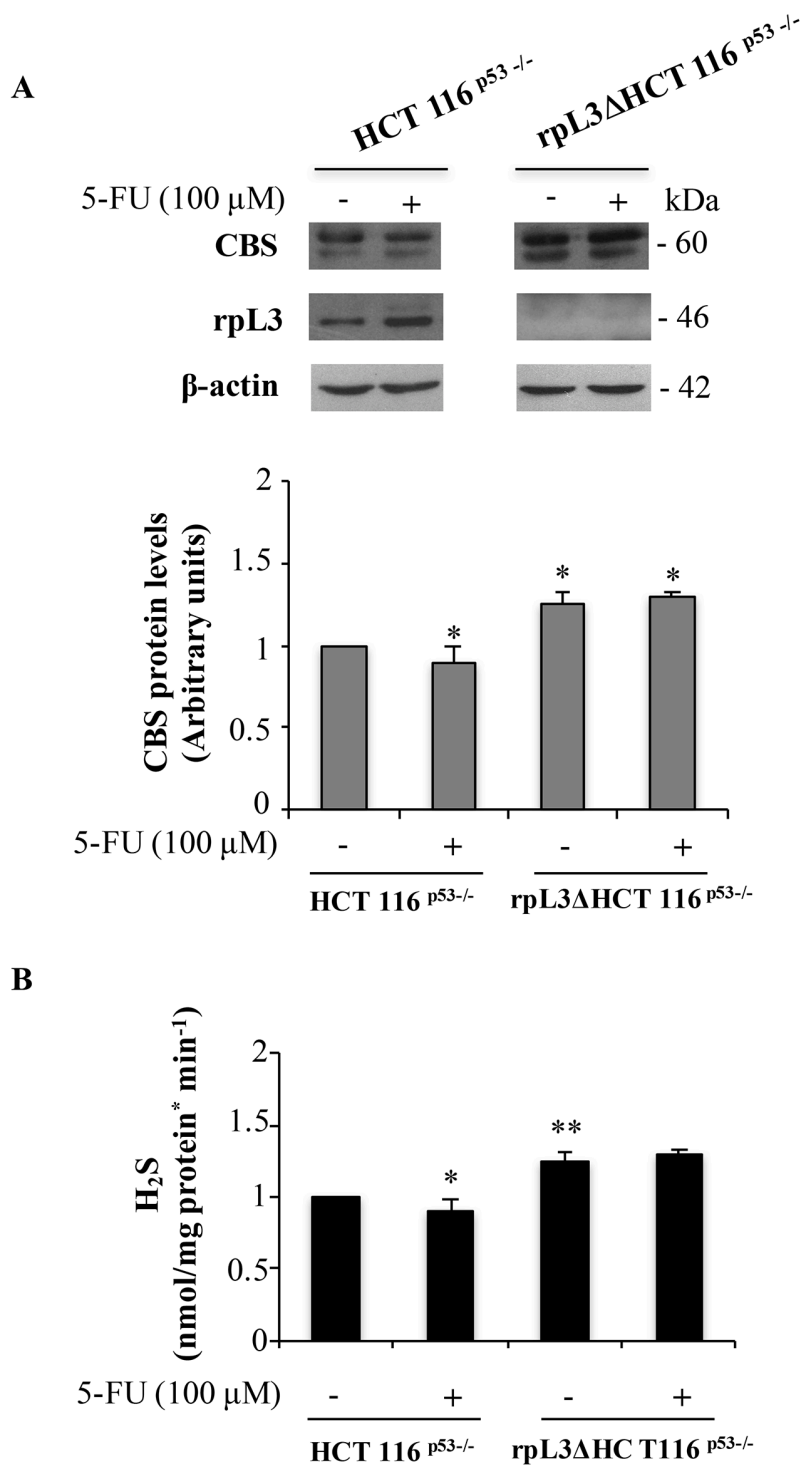
5-FU induced rpL3 down-regulates CBS expression levels and H2S production **A.** Western blotting of rpL3 and CBS protein expression. HCT 116^p53−/−^ and rpL3Δ HCT 116^p53−/−^ cells were treated or not with 100 μM 5-FU for 24 h. Protein extracts from the samples were analyzed by western blotting with antibodies against rpL3 and CBS. β-actin was used as loading control. Quantification of signals is shown. *P < 0.05 vs. untreated HCT 116^p53−/−^ cells. **B.** Production of H_2_S from the same samples. **P < 0.01,*P < 0.05 vs. untreated HCT 116^p53−/−^ cells. Results illustrated in Figure 1–7 are representative of three independently performed experiments.

The Figure 1A shows that in HCT 116^p53−/−^ cells 5-FU treatment caused the up-regulation of rpL3 expression levels, as previously demonstrated [9], coupled to the down-regulation of CBS protein levels. The observed CBS decrease was associated to a significant reduction of H_2_S biosynthesis (Figure 1B). Of note, alterations of CBS protein amounts were also observed upon 5-FU treatment in condition of rpL3 depletion. In fact, densitometric analysis of western blots revealed that the loss of rpL3 was associated to a significant increase of CBS expression levels with comparable higher production of H_2_S (Figure 1A, B).

These results indicate that the effect of 5-FU on CBS expression occurs through a molecular mechanism specifically mediated by rpL3.

### rpL3 regulates CBS expression at transcriptional level

**Figure 2:**
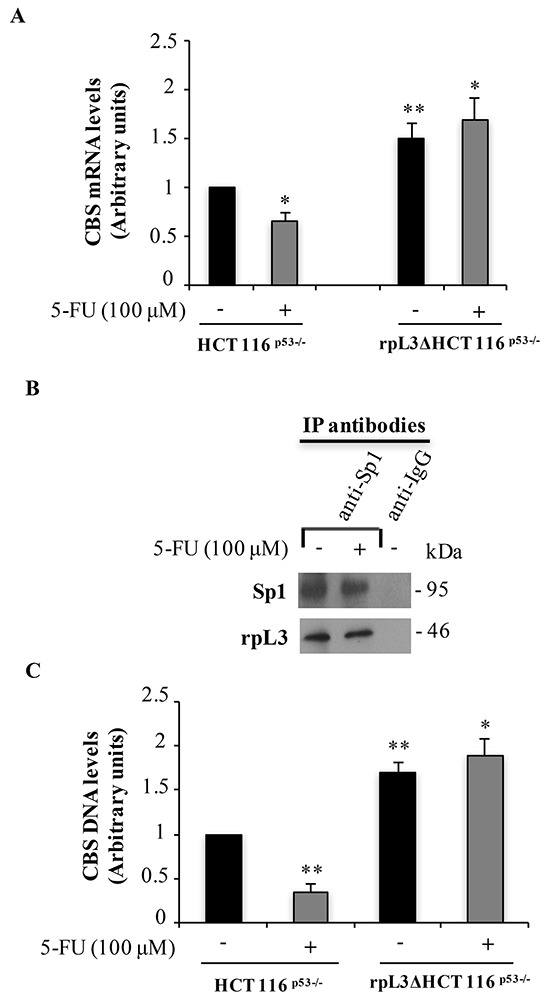
Upon 5-FU treatment, the interaction of rpL3 with Sp1 impairs its binding to CBS promoter and leads to a decrease of CBS mRNA levels **A.** Total RNA from HCT 116^p53−/−^ cells untreated or treated with 100 μM 5-FU for 24 h was subjected to qPCR with primers specific for CBS mRNA. Quantification of signals is shown. **P < 0.01,*P < 0.05 vs. untreated HCT 116^p53−/−^ cells. **B.** Analysis of the interaction between rpL3 and Sp1. Protein samples of Sp1 or IgG immunocomplexes from HCT 116^p53−/−^ cells untreated or treated with 100 μM 5-FU for 24 h were analyzed by western blotting with antibodies against Sp1 and rpL3. Note the absence of signal in IgG immunocomplex. **C.** Analysis of the interaction between Sp1 and CBS gene promoter. DNA-Sp1 or DNA-IgG immunocomplexes from HCT 116^p53−/−^ and rpL3ΔHCT 116^p53−/−^ cells, untreated or treated with 100 μM 5-FU for 24 h, were analyzed by qPCR with primers specific for CBS gene promoter. Quantification of the signals is shown. **P < 0.01,*P < 0.05 vs. untreated HCT 116^p53−/−^cells.

We have recently demonstrated that, after drug induced nucleolar stress, the ribosome-free rpL3 was able to act as transcriptional factor [8–10]. Consequently we became interested to determine whether the rpL3-mediated regulation of CBS protein levels was consequent to the rpL3 control on the activity of CBS promoter. To this aim, total RNA from untreated and 5-FU treated HCT 116^p53−/−^ and rpL3ΔHCT 116^p53−/−^ cells was isolated and CBS mRNA levels were quantified by using RT-qPCR. We found a decrease (about 40%) in CBS mRNA levels upon 5-FU treatment in HCT 116^p53−/−^ cells (Figure 2A). Of note, the rpL3 loss and 5-FU treatment cooperate to produce a strong increase of CBS mRNA amounts. These results suggest a role of rpL3 as regulatory factor of CBS transcription. The expression of CBS gene is mainly regulated, at transcriptional level, by Sp1 [16] and we have previously demonstrated that rpL3 interacts with Sp1 [8]. In order to verify if a specific interaction between these two proteins also occurred upon 5-FU induced nucleolar stress, HCT 116^p53−/−^ cells untreated or treated with 100 μM 5-FU for 24 h, were collected and protein extracts subjected to immunoprecipitation experiments by using anti-Sp1 and anti-IgG as control. Immunoprecipitated proteins were separated by SDS-PAGE and the presence of Sp1 and rpL3 was investigated in the immunoprecipitated complexes by western blotting. The Figure 2B shows that in untreated cells rpL3 interacted with Sp1 as previously demonstrated [8]. A specific binding of rpL3 with Sp1 was also observed after 5-FU treatment.

To gain insights into the mechanism by which rpL3 was associated to reduction of CBS mRNA levels and in order to understand whether rpL3-Sp1 interaction played a role in CBS transcription, we studied the effects of alterations in rpL3 intracellular levels on Sp1 binding to the CBS promoter by performing ChIP experiments. We specifically immunoprecipitated Sp1 from HCT 116^p53−/−^ and rpL3ΔHCT 116^p53−/−^ cells treated with 100 μM 5-FU for 24 h. Results from qPCR assays on the samples indicated that in untreated cells, the transcriptional activator Sp1 binds CBS promoter (Figure 2C), as previously demonstrated [16]. In 5-FU treated cells, the binding of Sp1 on CBS promoter was significantly decreased compared to that observed in the control (Figure 2C). Noteworthy, in rpL3 depleted cells either 5-FU treated or untreated, the interaction of Sp1 with CBS promoter was strongly increased, indicating a reorganization of the protein complexes on the promoter. These data imply that the interaction of Sp1 with CBS promoter is affected by rpL3 status.

### rpL3 binds CBS *in vivo* and controls CBS stability

**Figure 3:**
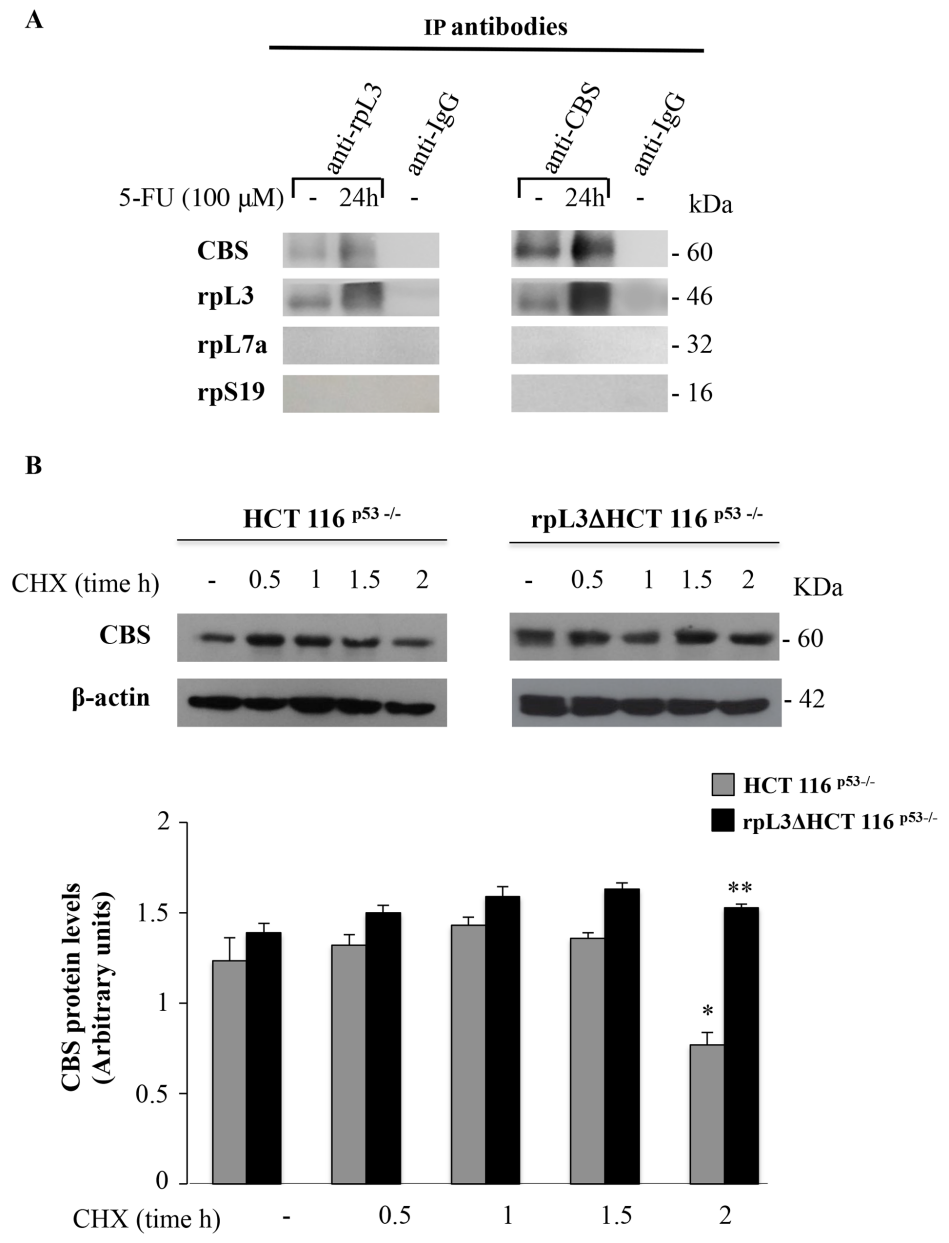
rpL3 interacts with CBS and negatively affects its half-life **A.** *In vivo* binding of rpL3 and CBS. rpL3 or CBS were specifically immunoprecipitated from cell extracts with antibodies against the endogenous rpL3 and CBS. Immunoprecipitates were separated by SDS–PAGE and immunoblotted with antibodies versus the indicated proteins. Note the absence of signal in IgG immunocomplex. **B.** HCT 116^p53−/−^ and rpL3ΔHCT 116^p53−/−^ cells were treated with CHX for 0.5, 1, 1.5 and 2 h. After the treatment, cell lysates were prepared and immunoblotted with anti-CBS. β-actin was used as control. Quantification of the signals is shown. *P < 0.05 vs. untreated HCT 116^p53−/−^cells; ^##^P < 0.01 vs. HCT 116^p53−/−^ cells treated with CHX for 2h.

We next investigated the possibility that rpL3 and CBS could associate *in vivo*. To this aim, HCT 116^p53−/−^ cells were treated with 100 μM 5-FU for 24 h. Then, rpL3 and CBS were specifically immunoprecipitated from cell extracts by using antibodies against the endogenous proteins. Immunoprecipitated proteins were separated by SDS-PAGE and analyzed by western blotting with anti-rpL3, anti-CBS and as controls anti-rpL7a and anti-rpS19 two arbitrary proteins of large and small subunit, respectively. The results of these experiments showed that rpL3 and CBS were co-immunoprecipitated thus indicating that they can associate in vivo, notably this interaction was strongly enhanced after 5-FU treatment (Figure 3A).

Furthermore, the absence of signal for rpL7a and rpS19 in both immunoprecipitates indicated that the free rpL3, not associated into ribosome, is able to interacts with CBS. A control immunoprecipitate obtained with anti-IgG antibodies did not give any signal when probed with the same antibody.

To verify whether the interaction of rpL3 and CBS affected CBS turnover and, in particular, to determine whether rpL3 was essential to maintain CBS intracellular abundance, we examined the level of CBS in HCT 116^p53−/−^ and rpL3Δ HCT 116^p53−/−^ cells. To this aim, cells were incubated with cycloheximide for various times (0.5, 1, 1.5, 2 h). After the incubation, cells were harvested, lysated and the level of CBS was determined by western blot analysis. The results illustrated in Figure 3B demonstrate that the half-life of CBS was greater in cells upon rpL3 silencing. All together these data indicate that rpL3 physically interacts with CBS and induces its degradation.

### Role of rpL3 in mitochondrial apoptosis upon 5-FU treatment

The induction of apoptosis is a standard strategy used in anticancer therapy [18]. Recently, we have shown a proapoptotic function of rpL3 [9], and other authors have demonstrated that the downregulation of CBS triggers mitochondrial apoptosis [17]. In order to investigate the factors leading to the rpL3-induced apoptosis in HCT 116^p53−/−^ cells upon 5-FU treatment, we primarily determined the effect of 5-FU-induced free rpL3 on activities of apoptosis-related proteins of the mitochondria-mediated pathway. This pathway contains several members, including the anti-apoptotic protein, Bcl-2 and the apoptosis-promoting protein, Bax. The ratio Bcl-2/Bax is used to evaluate the occurrence and severity of apoptosis [18]. To this aim, HCT 116^p53−/−^ and rpL3Δ HCT 116^p53−/−^ cells were treated with 5-FU 100 μM for 24 h. Then, cells were lysated and protein extracts were analysed by western blotting for the expression profile of procaspase-3, Bcl-2 and Bax.

**Figure 4:**
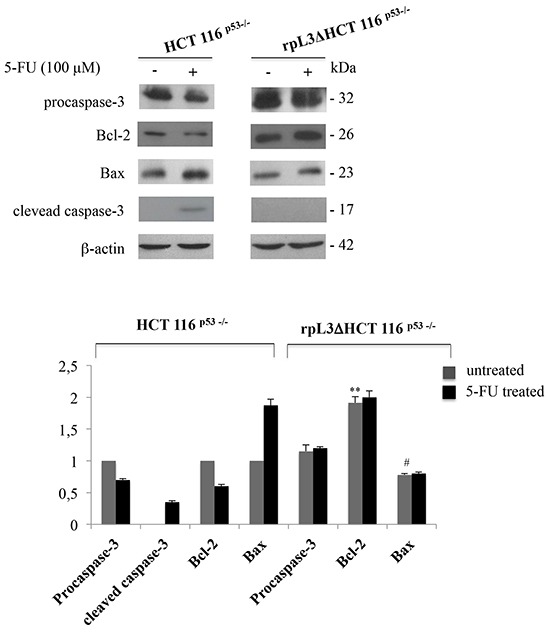
rpL3 activates mitochondrial apoptosis upon 5-FU treatment Representative western blotting of procaspase-3, Bcl-2, Bax and active clevead caspase-3 protein expression. HCT 116^p53−/−^ and rpL3ΔHCT 116^p53−/−^ cells were treated or not with 100 μM 5-FU for 24 h. After the treatment, protein extracts from the samples were analyzed by western blotting with the indicated antibodies. β-actin was used as control. Quantification of the signals is shown. **P < 0.01 vs. Bcl-2 in untreated HCT 116^p53−/−^ cells; ^#^ P < 0.05 vs. Bax in untreated HCT 116^p53−/−^cells.

The Figure 4 shows a marked decrease of Bcl-2 levels associated to an increase of Bax amounts in HCT 116^p53−/−^ cells treated with 5-FU as compared to untreated cells. These data are in good correlation with the apoptosis induced in HCT 116^p53−/−^ cells by 5-FU. Of interest, these effects were not obeserved when we treated rpL3ΔHCT 116^p53−/−^ cells with 5-FU.

Caspase apoptosis proteins are activated when the ratio Bcl-2/Bax is reduced [19], therefore we analyzed the expression of caspase-3 which is a key regulator in the caspase-dependent cell apoptosis pathway [20, 21]. Consistent with the higher apoptotic ability of the treated cells, the presence of the active caspase-3 form, which is proteolytically generated during apoptosis, was observed. These findings add strength to the hypothesis that the involvement of a caspase-dependent pathway through a caspase-3-triggered mitochondrial pathway may lead to rpL3-mediated apoptosis.

### rpL3 triggers CBS translocation to mitochondria upon 5-FU treatment

**Figure 5:**
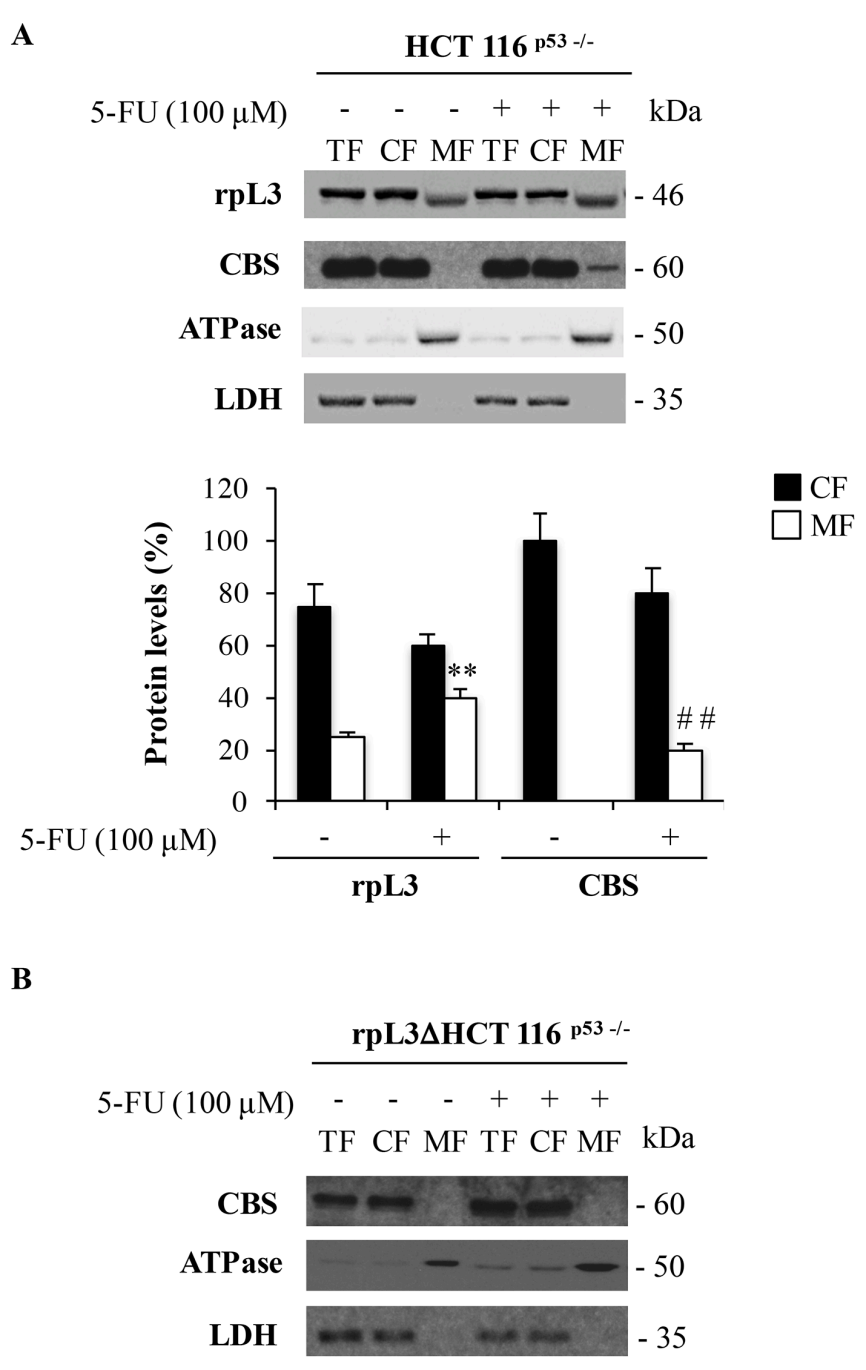
5-FU induced rpL3 triggers CBS mitochondrial translocation **A.** HCT 116^p53−/−^ and **B.** rpL3Δ HCT 116^p53−/−^ cells were treated or not with 100 μM 5-FU for 24 h. After the treatment, cells were subjected to fractionation to obtain the cytosolic fraction (CF) and the mitochondrial fraction (MF). Protein extracts from the samples were analyzed by western blotting with antibodies against rpL3 and CBS. LDH and ATPase were used as controls for CF and MF, respectively. Quantification of the signals is shown. **P < 0.01 vs. rpL3 in untreated MF; ##P < 0.01 vs. CBS in untreated MF.

In order to investigate the sorting of rpL3 to specific subcellular regions in condition of 5-FU-induced nucleolar stress, we used a subcellular fractionation procedure. Using biochemical techniques, we isolated from HCT 116^p53−/−^ cells two distinct fractions corresponding to the cytosolic fraction (CF) and the mitochondrial fraction (MF). These two fractions were assayed for lactate dehydrogenase (LDH), a marker component of the CF [22], and ATPase as control of the MF. Since LDH was recovered exclusively in the cytosolic fraction (Figure 5A), the mitochondrial fraction appeared to be free of soluble fraction contaminants. As expected, the signal of ATPase occurred predominantly in the MF (Figure 5A).

In untreated cells, rpL3 was significantly more abundant in the CF but it was also detected in the MF. Specifically, the quantitative analysis revealed that about 75% of rpL3 was associated with the CF fraction and 25% in MF (Figure 5A). Of note, in 5-FU treated cells, the association of rpL3 to the mitochondria was more pronounced compared to that observed in untreated cells (40% vs 25%). Analysis of subcellular distribution of rpL7a and S19, two arbitrary proteins of large and small subunit respectively, between CF and MF of 5-FU treated cells by western blotting shows that rpL7a and S19 are not present in the MF, indicating that the rpL3 localizes into the mithocondrion as form not associated to ribosome (Supplementary Figure S1).

The analysis of the distribution of CBS in the CF versus the MF revealed that in untreated cells, CBS was exclusively detected in the CF (Figure 5A). The induction of nucleolar stress mediated by the treatment of cells with 5-FU affected the distribution of CBS in the distinct pools. Quantification of the signal for CBS showed that, after 5-FU treatment, about 16% of the signal occurred in the MF (Figure 5A). These findings clearly indicate a specific association of rpL3 and CBS with the mitochondria during drug-induced nucleolar stress.

Next, in order to verify whether the shift of CBS from the CF to the MF was mediated by rpL3, the CBS mitochondrial content was evaluated also in rpL3ΔHCT 116^p53−/−^ cells. Western blotting analysis of biochemical fractions from these cells after 5-FU treatment showed the absence of the signal for CBS in MF fraction (Figure 5B). These data strongly indicate that rpL3 was essential in mediating the association of CBS to mitochondria in condition of nucleolar stress.

### rpL3 mediates cytochrome c release after 5-FU treatment

**Figure 6:**
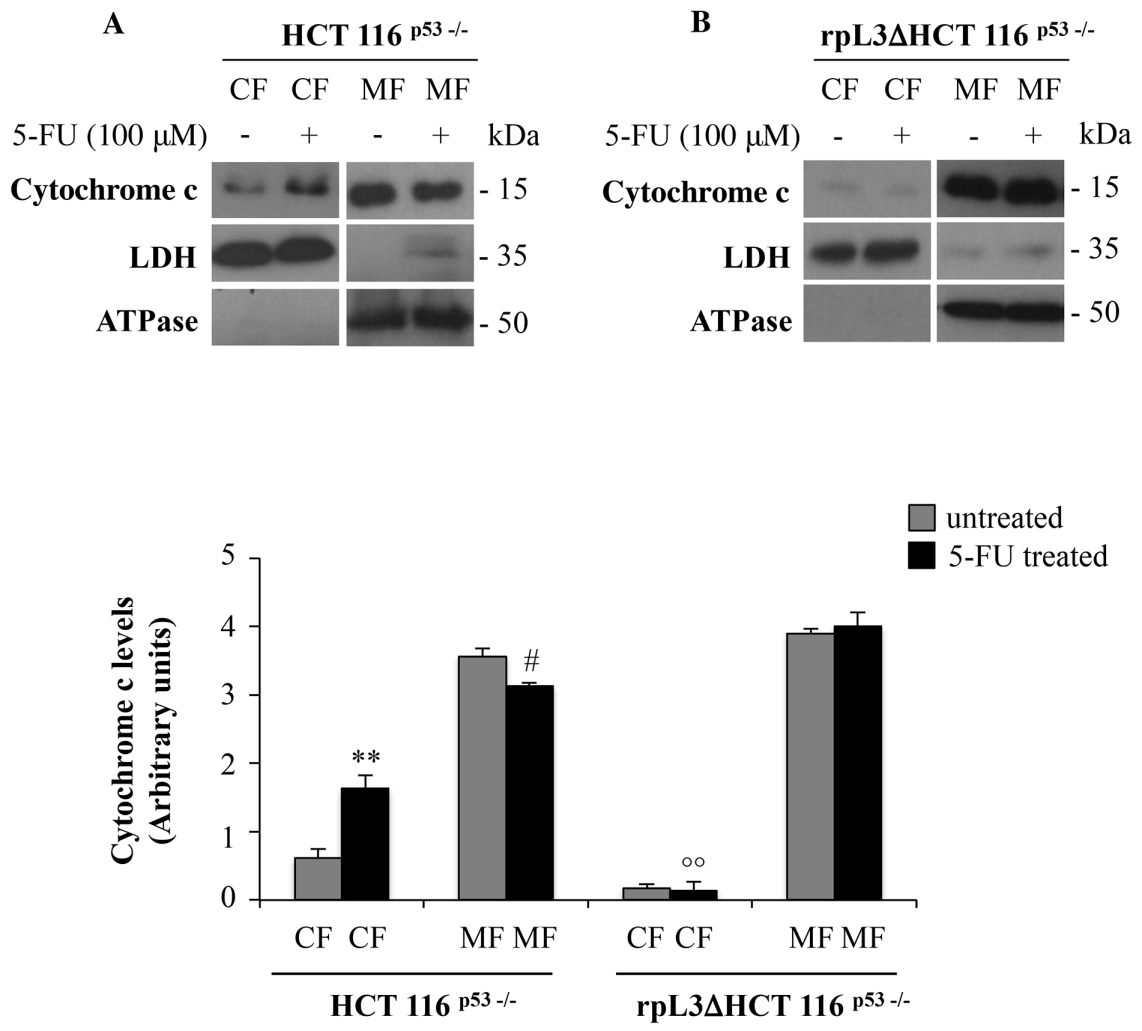
rpL3 translocation upon 5-FU treatment mediates the release of cytochrome c from mitochondria Representative western blotting of cytochrome c protein expression. **A.** HCT 116^p53−/−^ and **B.** rpL3Δ HCT 116^p53−/−^ cells were treated or not with 100 μM 5-FU for 24 h. After the treatment, cells were subjected to fractionation to obtain the cytosolic fraction (CF) and the mitochondrial fraction (MF). Protein extracts from the samples were analyzed by western blotting with antibodies against cytochrome c. LDH and ATPase were used as controls for CF and MF, respectively. Quantification of the signals is shown. **P < 0.01 vs. cytochrome C in CF from untreated HCT 116^p53−/−^ cells; #P < 0.05 vs. cytochrome C in MF from untreated HCT 116^p53−/−^ cells; °° P < 0.01 vs. cytochrome C in MF from 5-FU treated HCT 116^p53−/−^ cells.

Release of cytochrome c from the mitochondria to the cytosol is a critical step in apoptotic cell death [23], thus we became interested to examine cytochrome c release to the cytosol in HCT 116^p53−/−^ and rpL3ΔHCT 116^p53−/−^ cells upon 5-FU treatment. To this aim, HCT 116^p53−/−^ and rpL3ΔHCT 116^p53−/−^ cells were subjected to biochemical fractionation to isolate the CF and MF. Fractionation efficiency was evaluated by using LDH and ATPase as control of the CF and MF, respectively. Figure 6A shows that in untreated HCT 116^p53−/−^ cells, a significant amount of cytochrome c was detected in the MF. 24 h after 5-FU treatment, a redistribution of cytochrome c between the subcellular compartments was observed. In fact, in this condition the decrease of cytochrome c in MF was associated to an increase of it in CF. Of interest, in rpL3ΔHCT 116^p53−/−^ cells the treatment with 5-FU was not associated to cytochrome c redistribution betwen CF and MF (Figure 6B).

### CBS regulates migration and invasion of colon cancer cells upon 5-FU treatment

In order to confirm the relevance of CBS in the cell response to 5-FU, we decided to perform gene silencing experiments. To this end, we generated a cell line stably silenced for CBS derived from HCT 116^p53−/−^ cells namely CBSΔHCT 116^p53−/−^, in which CBS protein expression was abrogated using shRNA technology.

**Figure 7:**
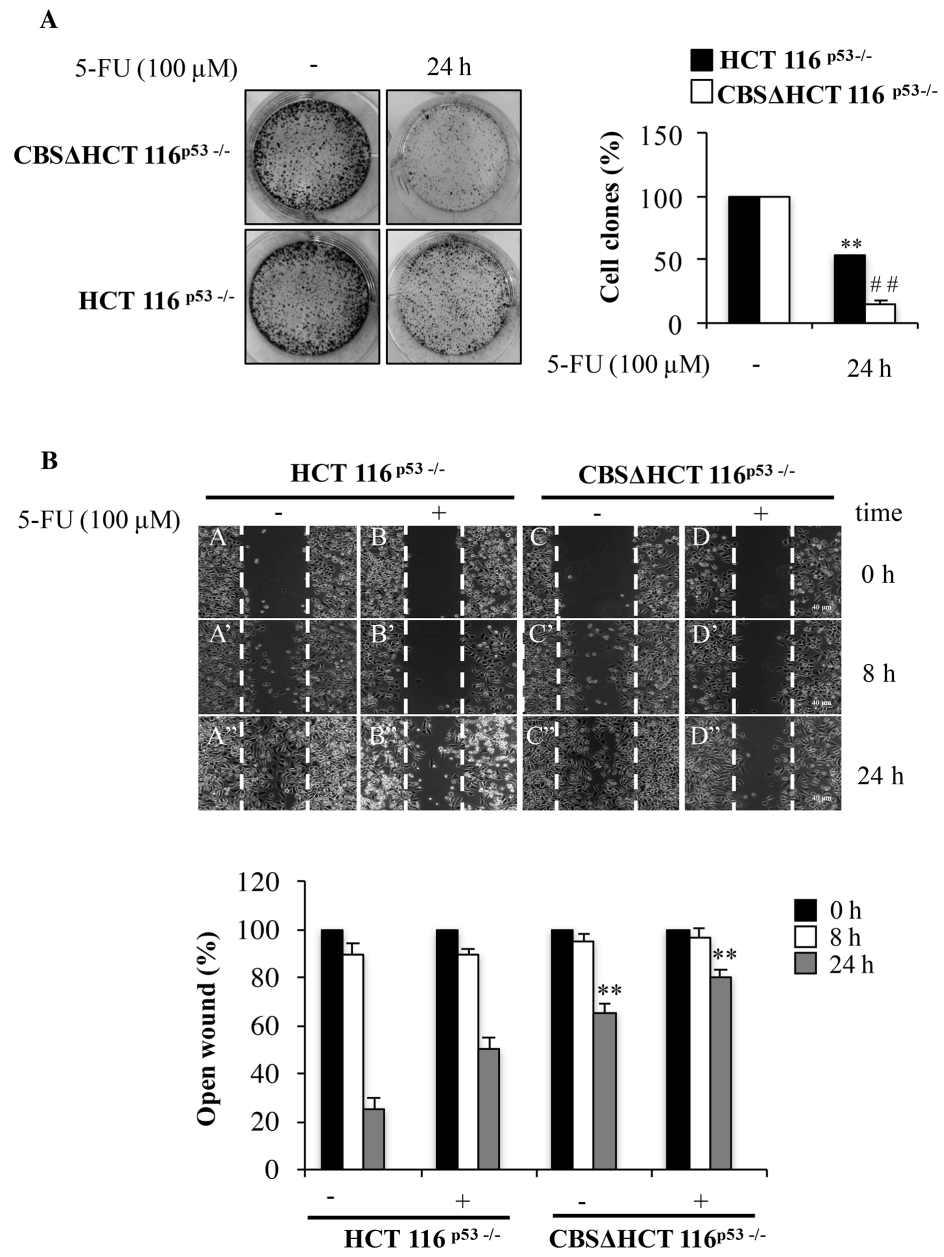
Depletion of CBS decreases cell proliferation and migration **A.** Representative images of clonogenic analysis for cell proliferation in HCT 116^p53−/−^ and CBSΔHCT 116^p53−/−^ cells after 5-FU treatment. **P < 0.01 vs. untreated HCT 116^p53−/−^ cells; ##P < 0.01 vs. untreated rpL3Δ HCT 116^p53−/−^cells. **B.** Representative images of wound healing assay for cell proliferation in HCT 116^p53−/−^ and CBSΔHCT 116^p53−/−^ cells after 5-FU treatment. Wound widths were measured at 0, 8 and 24 h on 3 fields per well and averaged. Data is expressed as the fold-decrease of area respect to controls (A, B, C, D) set as 100%. **P < 0.01 vs untreated HCT 116^p53−/−^ cells.

Supplementary Figure S2 shows the decrease of CBS levels in different clones. We chosed the cell clone expressing the lowest level of CBS for further experiments. We examined the effect of CBS silencing in the cell response to 5-FU by a clonogenic assay. To this aim, HCT 116^p53−/−^ and CBSΔHCT 116^p53−/−^ cells were treated with 5-FU for 24 h. Figure 7A shows that in HCT 116^p53−/−^ cells, the colony number was reduced upon exposure to 5-FU thus confirming the ability of the drug to inhibit clonogenicity. It is noteworthy that in CBSΔHCT 116^p53−/−^ cells the colony-forming activity upon 5-FU treatment was significantly reduced compared to the colony-forming activity of untreated cells (Figure 7A). These results suggest that the loss of CBS plays a critical role in cell response to 5-FU.

Moreover, we investigated the effect of CBS silencing on cell motility in HCT 116^p53−/−^ cells upon 5-FU exposure. Cell migration was determined using wound healing assay and quantitatively evaluated in terms of occupation rate of open wound (see Materials and Methods). As indicated in Figure 7B, the wound healing ability of 5-FU treated HCT 116^p53−/−^ cells was reduced in time dependent manner compared to that observed in untreated cells. Likewise, the quantitative analysis showed that the open wound of 5-FU treated HCT 116^p53−/−^ cells was decreased of about 10% and 50% compared to untreated cells after 8 and 24 h respectively. When CBS was depleted, the wound healing ability of 5-FU treated HCT 116^p53−/−^ cells was markdely reduced, demonstrating that CBS depletion was able to furher decrease 5-FU-reduced cell motility. In particular, the quantification indicated that the open wound of 5-FU treated CBSΔHCT 116^p53−/−^ cells was decreased of about 10% and 20% after 8 and 24 h, respectively (Figure 7B).

All together these findings indicate that CBS inhibition promoted chemosensitization, decreased colony-forming potential in clonogenic assays and cell migration.

## DISCUSSION

It is known that more than 50% of human cancers lack functional p53 [24]. Consequently, drugs triggering cell death in p53-null cells may have great potential in the treatment of many cancers. 5-FU is a drug able to induce nucleolar stress [4]. Recently, we have demonstrated that after nucleolar stress induced by 5-FU treatment in colon cancer cells lacking active p53, rpL3 is up-regulated and accumulated as ribosome free form needed to mediate 5-FU apoptotic cell response. In fact the loss of rpL3 makes chemotherapeutic drug ineffective [9]. Moreover, we have demonstrated that overexpression of rpL3 in cancer cells laking p53 induces cell cycle arrest or apoptosis by positively modulating the activity of p21 [8].

These finding strongly suggest that the knowledge of rpL3 status in relation to p53 status in colon cancers may have a significant value in terms of the efficacy of chemotherapy. The focus of the current study was to increase the knowledge on the relationship between alteration on rpL3 levels induced by nucleolar stress and activity of 5-FU for the treatment of the colon cancers laking p53; the full understanding of p53-independent pathways implicated in colon cancer molecular biology may provide new targets for cancer therapy.

The colon is known to synthesize and metabolize H_2_S and to be exposed to exogenous H_2_S as consequence of metabolic activity of luminal microbiota [25]. There are evidences supporting both the cytoprotective and cytotoxic effects of H_2_S [26]. Recent data demonstrate that H_2_S is implicated in human colorectal cancer development [27]. In addition, it has been recently reported a functional relation between H_2_S production and p21 expression in colon cancer. In particular, an exogenously administered H_2_S donor, NaHS, inhibited significantly p21 expression leading to colon cancer cell proliferation [28]. All these findings prompted us to explore the hypothesis of a new rpL3 dependent stress response pathway involving CBS. Specifically, we wondered whether CBS was involved in rpL3-mediated stress pathway upon 5-FU treatment in p53 null colon cancer HCT 116^p53−/−^ cells. 5-FU treatment of HCT 116^p53−/−^ cells caused a reduction in CBS expression levels and H_2_S production. Conversely, a marked increase of CBS expression and H_2_S biosynthesis was observed upon rpL3 silencing and 5-FU treatment indicating that the alteration in CBS and H_2_S levels after drug treatment is rpL3-dependent (Figure 1). According to this, the analysis of cell cycle distribution of HCT 116^p53−/−^ cells stably depleted of rpL3 led us to exclude that observed effects are consequence of cell cycle alterations (Supplementary Figure S3). In addition, unchanged expression levels of other ribosomal proteins in this cell line indicate that the obtained results are not due to ribosome deficiency (Supplementary Figure S4).

Analysis of CBS mRNA levels and ChIP experiments in HCT 116^p53−/−^ cells and rpL3ΔHCT 116^p53−/−^ cells, untreated or treated with 5-FU, imply a negative role of rpL3 in CBS gene transcription (Figure 2). It is well-known that CBS promoter contains Sp1 binding sites and that Sp1 is essential for its transactivation [16]. Interestingly, rpL3 associates with Sp1 and this association is necessary for rpL3-mediated p21 promoter transactivation [8]. Here, we demonstrate that rpL3 associates *in vivo* with Sp1 in condition of 5-FU treatment (Figure 2B). These findings led us to hypothesize that the binding of Sp1 to CBS promoter could be influenced by rpL3 levels; specifically we propose that rpL3 behaves as a negative regulatory factor of CBS transcription through its binding to Sp1. The formation of the complex rpL3-Sp1 could induce conformational changes in Sp1 promoting its release from the CBS promoter. Analysis of immunoprecipitate of rpL3 and CBS in HCT 116^p53−/−^ cell extracts treated or not with 5-FU showed that rpL3 and CBS coimmunoprecipitate together indicating that these proteins associate *in vivo* (Figure 3A). The stability of an enzyme might play an important role in the regulation of its activity. Here we demonstrated that rpL3 is able to reduce CBS protein stability (Figure 3B). All together these results demonstrate that ribosome free rpL3 reduces CBS protein levels after 5-FU treatment in colon cancer cells devoid of p53 by acting at both transcriptional and post-translational levels.

CBS protein does not contain a NH_2_ terminal signal to direct it to the mitochondria [29]. However, in the present study we show a 5-FU induced mitochondrial CBS traslocation in HCT 116^p53−/−^ cells (Figure 5A). In this process rpL3 plays a crucial role as in rpL3ΔHCT 116^p53−/−^ cells 5-FU treatment failed to direct CBS into the mitochondria (Figure 5B). Previous studies have shown that Lon protease, a major degradation enzyme in mithocondria recognizes and degrades CBS enzyme [29]. Starting from these findings we hypothesize that rpL3 mediated translocation of CBS into the mitochondrion is associated to its degradation via Lon protease.

Cytochrome c is normally located in the intermembrane space of the mitochondrion, loosely bound to the inner membrane [30]. Although apoptosis can occur *via* cytochrome c-independent mechanisms, it is well established that release of cytochrome c into the cytosol results in caspase-3 mediated activation of apoptosis [31]. Here we show a significant release of cytochrome c in the cytosol of 5-FU treated HCT 116^p53−/−^ cells (Figure 6) coupled to a significant increase in active cleaved caspase-3 and a decrease in the Bcl-2/Bax ratio (Figure 4). All these data are consistent with an increase of apoptosis induced upon 5-FU treatment. Most notably, silencing of rpL3 in HCT 116^p53−/−^ cells completely abolished these effects. Indeed, in these cells we observed a significant increase in Bcl-2 levels, and 5-FU treatment did not modify mitochondrial Bax levels. This resulted in an increase in the Bcl-2/Bax ratio, a response that would be protective against apoptosis. Consistent with this, in the cytosol of 5-FU treated rpL3ΔHCT 116^p53−/−^ cells there was lack of release of citochrome c. All togheter these results indicate that rpL3 is a major player in the apoptotic activity of 5-FU.

In order to further characterize the role of CBS in rpL3-mediated cell response to 5-FU, we stably silenced CBS in HCT 116^p53−/−^ cells.

CBS inhibition did not cause alteration of cell cycle distribution (Supplementary Figure S3) but specifically promoted chemosensitization, in fact we observed a decreased colony-forming potential in clonogenic assays and inibition of cell migration (Figure 7).

**Figure 8:**
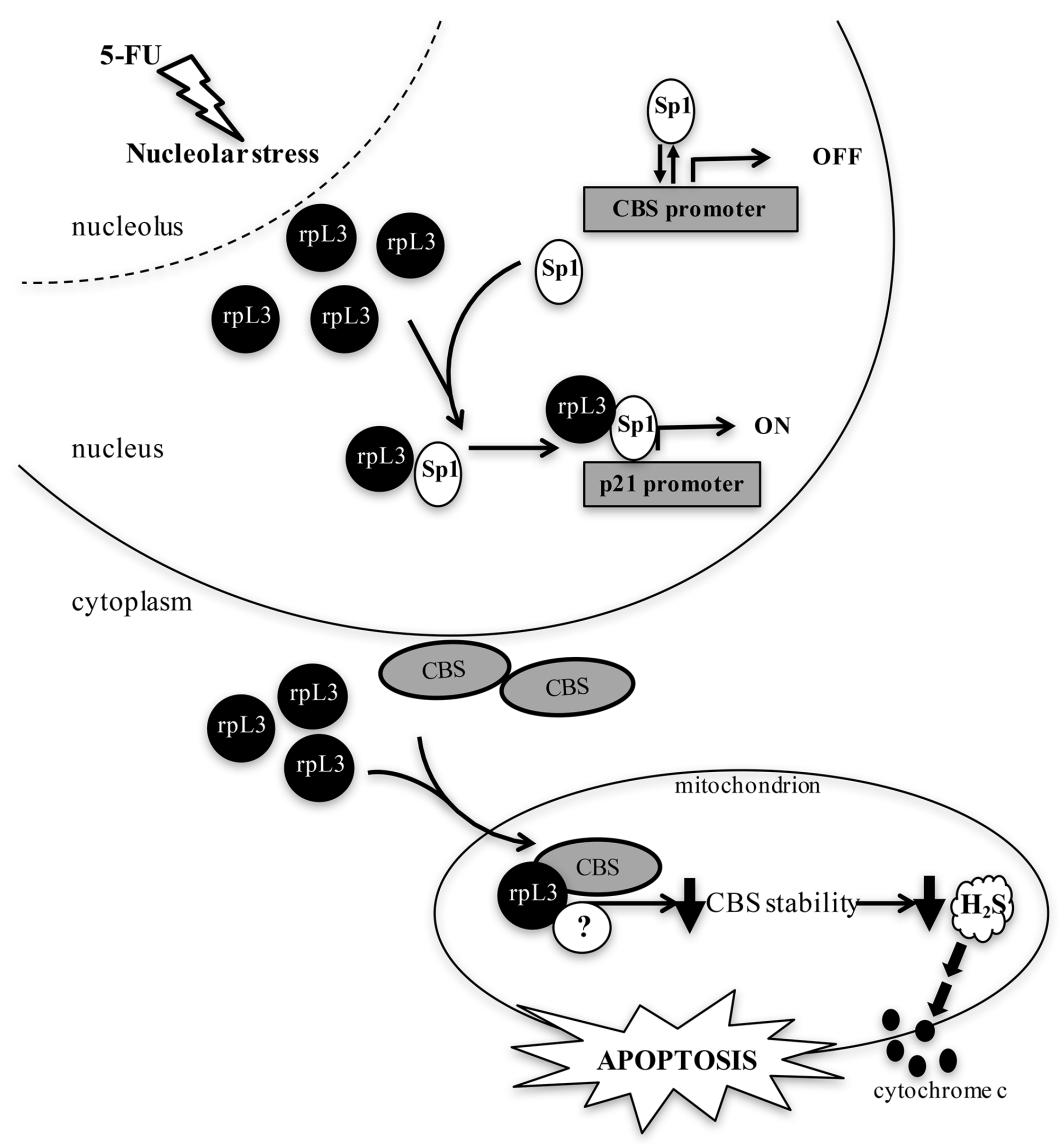
Schematic representation of proposed model 5-FUinduced nucleolar stress caused an induction of rpL3 as ribosome-free form. In response to 5-FU, ribosome free rpL3 becomes a regulator of CBS expression. At transcriptional level, rpL3 binds Sp1 and causes its release from CBS promoter. At the same time, rpL3 could recruit Sp1 on p21 promoter to induce its trans-activation At post-translational level, rpL3 binds CBS and trigger it into the mitochondria for degradation. rpL3 effects associate to cytochrome c release, increase of Bax/Bcl2 ratio and caspase activation.

On the basis of these results, we propose a working model in which following 5-FU treatment, ribosome free rpL3 controls CBS expression at both transcriptional and post-translational levels. In the nucleus, under 5-FU induced nucleolar stress, the ribosome free rpL3 is able to activate p21 promoter [9–10] and to inhibit CBS transcription. We have previously demonstrated that Sp1 is a key component of rpL3-mediated p21 transactivation. Therefore it is likely that rpL3 may bind and sequester Sp1 from CBS promoter with consequent downregulation of CBS expression. At the same time, rpL3 could recruit Sp1 on p21 promoter thereby activating p21 expression (Figure 8).

In the cytoplasm, ribosome free rpL3 associates with CBS to shuttle it into the mitochondria. We hypothesyze that rpL3-CBS interaction leads to destabilization of mitochondrial CBS and, in turn, to a reduction in H_2_S biosynthesis. Several reports indicate an anti-apoptotic action of H_2_S [32]. Along this line, the decrease in H_2_S levels correlates with cytochrome c release from mitochondria, increased Bax/Bcl2 ratio and caspase activation (Figure 8).

In summary, we identified free rpL3 as a key regulator of CBS expression acting at transcriptional and post-translational levels. Taking into account the important role of CBS-derived H_2_S in tumor growth and proliferation it is evident that a fine regulation of CBS levels is required to prevent cancer as well as in response to chemotherapy. In the light of our findings, we suggest that the association of rpL3 mediated trascriptional and post-translational regulation of CBS levels might represent a way to fine-tune the amount of this protein to the appropriate levels. In other words, this fine regulation might provide a means with which to lower the threshold of CBS expression below what would otherwise be possible by modulating the transcription rate alone.

As a newly identified CBS repressor, rpL3 may be a potential cytotoxic agent against colon cancers lacking of p53. The relevance of these results suggests that therapeutic strategies aimed to upregulating rpL3 may be effective in the treatment of these tumors. Recently, we have developed novel polymeric nanoparticles based on a core of poly(lactic-co-glycolic) acid (PLGA) and a polymer shell of Hyaluronan (HA) and Polyethyleneimine (PEI) that represent a very promising system for the targeted delivery of drug combinations taking advantage of the shell and core properties [33]. At the present, the challenge is to use these nanoparticles as platform to deliver the conventional drug 5-FU and the proapoptotic protein rpL3 for the treatment of colon cancers lacking functional p53.

## MATERIALS AND METHODS

### Cell cultures, transfections and drug treatments

HCT 116^p53−/−^ cells and rpL3ΔHCT 116^p53−/−^ cells, derived from HCT 116^p53−/−^ cell line and stably silenced for rpL3, were cultured in Dulbecco's Modified Eagle's Medium (DMEM) with glutamax (Invitrogen, Carlsbad, California) supplemented with 10% fetal bovine serum (FBS), 2 mM L-glutamine, penicillin-streptomycin 50 U/ml and 0.5 μg/ml puromycin (Sigma-Aldrich).

CBSΔHCT 116^p53−/−^ cell line was obtained from HCT 116^p53−/−^ cells as previously reported [34]. Cells were transfected with 2 μg of different shRNA CBS plasmids (Sigma-Aldrich) by using Lipofectamin 2000 (Life Technologies) according to the manufacturer's instructions. Stable clones were selected in medium containing 1 mg/ml of Puromicin (Sigma-Aldrich) and assayed for the detection of CBS expression level by western blotting.

shRNA transfections were performed in cells as previously described [34].

Drug treatments were performed by adding to cells 100 μM 5-FU (Sigma-Aldrich, St. Louis, MO, USA), CHX

### Cell fractionation

To analyze the subcellular distribution of rpL3 and CBS cells were separated into Cytosolic fraction (CF) and Mitochondrial fraction (MF) using a modified protocol [35]. Briefly, cells from three 100-mm plate (2 × 107cells) were washed with icecold PBS 1X and treated with 900 μl of ice cold isolation buffer (0,1M TRIS-MOPS, 0,1M EDTA-Tris, 1M sucrose). The cell suspension was placed in a glass potter, stroked 40 times and centrifuged at 600xg for 10 min at 4°C. The supernatant obtained under these conditions was centrifuged at 7000xg for 10 min at 4°C and the recovered supernatant was referred to as the CF. The pellet was resuspended in 200μl of ice-cold isolation buffer and then centrifuged at 7000xg at 4°C for 10 min. The resulting pellet was resuspended in 50 μl of ice-cold isolation buffer and referred to as the MF.

### Immunoprecipitation and western blotting

Immunoprecipitation assay was performed as previously reported [36]. Briefly, 1 mg of HCT 116^p53−/−^ whole cell lysate was incubated with 30 μl of protein A/G agarose beads coated with 5 μg of anti-rpL3 (Primm, Milan, Italy) or anti-CBS (Santa Cruz Biotechnology) at 4 °C for 12 h. The beads were washed and boiled in the SDS sample buffer. The eluted proteins were loaded on 12% SDS-PAGE and detected by western blotting as previously reported [37, 38]. Aliquots of protein samples (30 μg) were resolved by 12% SDS-gel electrophoresis and transferred into nitrocellulose filters. The membranes were blocked in PBS, 0.1% Triton (Santa Cruz). The proteins were visualized with enhanced chemiluminescence detection reagent according to the manufacturer's instructions (Pierce, Rockford, Illinois).

The membranes were challenged with anti-rpL3 and anti-rpL7a (Primm, Milan, Italy), anti-Sp1, anti-CBS, anti-Bcl-2, anti-Bax, anti-caspase 3 and anti-β-actin, anti-rpS19 (Santa Cruz Biotechnology). Proteins were visualized with enhanced chemiluminescence detection reagent according to the manufacturer's instructions (Pierce, Rockford, Illinois).

### Chromatin immunoprecipitation and Flow cytometry

Chromatin immunoprecipitation asssay and Flow cytometry analysis were performed as previously reported [9].

### Clonogenic assay

For clonogenic assay, cells (4 × 10^3^ in 6-well multidishes) were plated in triplicate and treated with 5-FU (100 μM) for 24 h or not. After 10 days, colonies were stained with 1% methylene 490 blue in 50% ethanol as previously reported [10].

### Wound healing assay

Cell motility was assessed using a wound healing assay. Cells (1 × 10^6^ per well) were treated with 5-FU (100 μM) for 24 h or not. The confluent monolayer cells were then carefully wounded using a sterilized pipette tip. Monolayer cells were photographed at 0, 8 and 24 h with an objective 10X. Quantitative analysis of wound assay was performed by measuring the gap area. The gap area was defined by using ImageJ Software (National Institute of Health, USA). Data is expressed as the fold-decrease of area respect to controls set as 100%. Bars represent the mean of triplicate experiments; error bars represent the standard deviation.

### Statistical analysis

Error bars represent mean±SEM from n=3 biological replicates. Statistical comparisons were made by Student t-test and one-way ANOVA followed by Bonferroni's test for multiple comparisons. P <0.05 was considered significant, P <0.001 was considered highly significant.

## SUPPLEMENTARY MATERIALS FIGURES


