# Supplementary material for: 5-FU targets rpL3 to induce mitochondrial apoptosis via cystathionine-β-synthase in colon cancer cells lacking p53
Source: Oncotarget. 2016 Jul 2;7(31):50333–48. doi: 10.18632/oncotarget.10385 (PMC5226586; doi:10.18632/oncotarget.10385)
Supplement: Supplementary file 1 [file oncotarget-07-50333-s001.pdf]

5-FU targets rpL3 to induce mitochondrial apoptosis via cystathionine-β-synthase in colon cancer cells lacking p53

SUPPLEMENTARY FIGURES

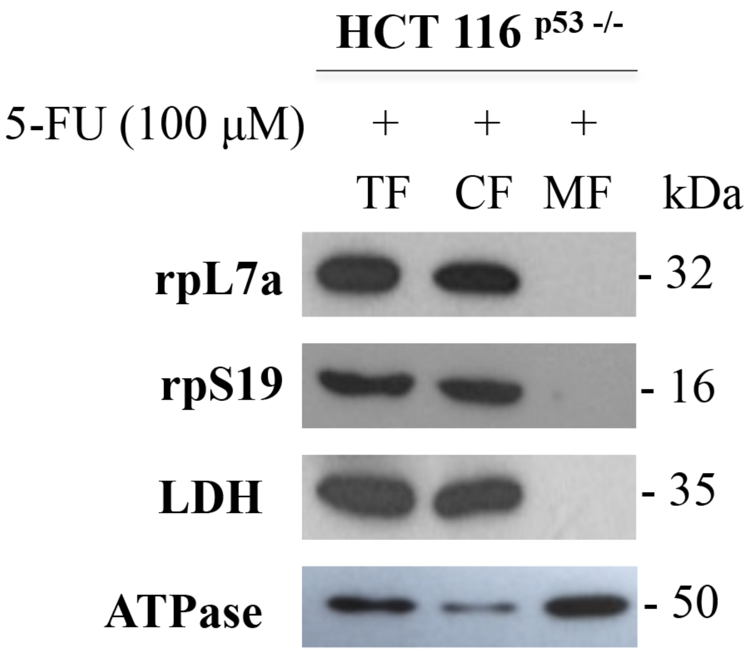

Supplementary Figure S1: Expression of rpL7a and rpS19 in mitochondrial fraction of HCT 116$^{p53-/-}$ cells after 5-FU treatment. HCT 116$^{p53-/-}$ cells were treated with 100 µM 5-FU for 24 h. Then, cells were subjected to fractionation to obtain the cytosolic fraction (CF) and the mitochondrial fraction (MF). Protein extracts from the samples were analyzed by western blotting with antibodies against rpL7a and rpS19. LDH and ATPase were used as controls for CF and MF, respectively.

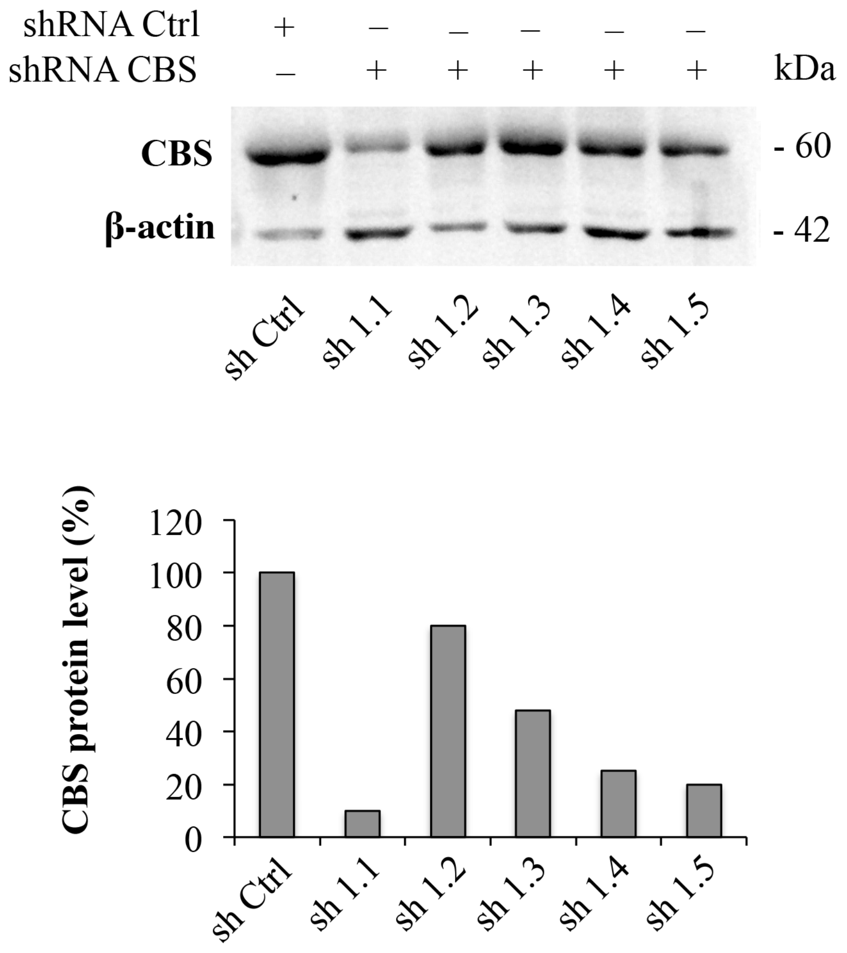

Supplementary Figure S2: Analysis of CBS expression levels in CBS depleted stable clones. Representative western blotting analysis of protein extracts from HCT 116$^{p53-/-}$ cells stably transfected with shRNAs specific for CBS (sh 1.1, sh 1.2, sh 1.3, sh 1.4, sh 1.5) or scrambled shRNA (shRNA Ctrl). Loading in the gel lanes was controlled by the detection of β-actin protein. Quantification of the signal is shown. CBS protein levels were calculated vs unsilenced cells set as 100 %.

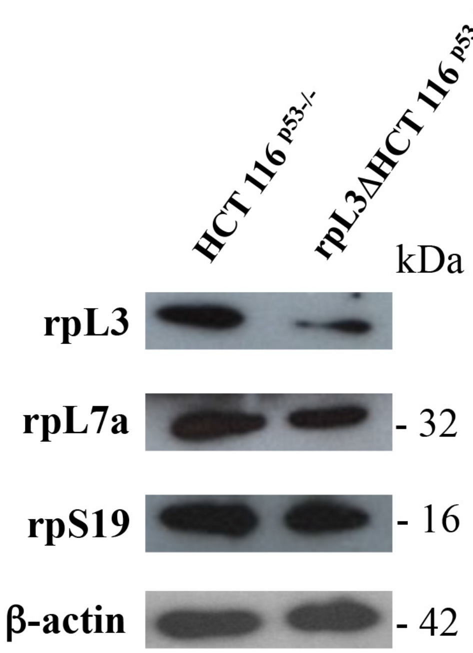

Supplementary Figure S3: Fluorescence-activated cell sorter (FACS) analysis of HCT 116$^{p53-/-}$, rpL3ΔHCT 116$^{p53-/-}$, CBSΔHCT 116$^{p53-/-}$ cells. Cells were stained with propidium iodide before analysis using FACSCalibur. Peaks representing histograms of cell numbers and table showing percentages in G1/G0, S, and G2/M phases are shown.

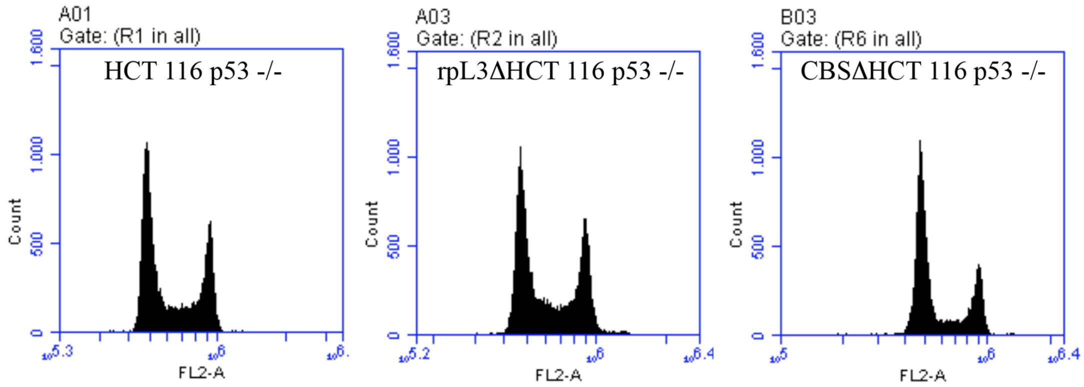

%	G0/G1	S	G2/M
HCT 116 p53 -/-	33,8± 0,34	13,7 ±0,22	26,1±0,40
rpL3ΔHCT 116 p53 -/-	39,4± 0,62	16,8±0,42	23,7±0,36
CBSΔHCT 116 p53 -/-	44,8± 0,91	9,6± 0,71	28,4± 0,83

Supplementary Figure S4: Analysis of rpL3, rpL7a and rpS19 expression in HCT 116$^{p53-/-}$ and rpL3ΔHCT 116$^{p53-/-}$ cells. Protein extracts from cells were analyzed by western blotting using antibodies against the indicated proteins. β-actin was used as loading control.
